# Chronic Pulmonary Histoplasmosis Identified in a Young Patient with Selective Immunoglobulin M Deficiency

**DOI:** 10.1155/2018/8740204

**Published:** 2018-05-20

**Authors:** Ania Preneta, Khaled M. Nada, Asima Raja, Moiz Kasubhai

**Affiliations:** Lincoln Medical and Mental Health Center, Bronx, NY, USA

## Abstract

Chronic histoplasmosis is typically diagnosed in patients who are immunocompromised or severely debilitated and who either live in or who have travelled to endemic areas. We report the case of a young, otherwise immunocompetent male patient who presented to a New York hospital with lobar consolidation and was found to have chronic pulmonary histoplasmosis. He described no history of travel to an endemic area. Immunological workup later revealed selective immunoglobulin M (IgM) deficiency. The literature has suggested a link between IgM deficiency and fungal infections. Recent research has also proposed a link between autoimmunity and IgM deficiency. Our clinical vignette describes the case of a patient with selective IgM deficiency who was diagnosed with pulmonary histoplasmosis without any clinical evidence of autoimmune disease.

## 1. Introduction

Histoplasmosis is extremely rare in presumed immunocompetent patients who do not reside in endemic areas [1]. This is a case of a young male who presented with right middle lobe pneumonia, later diagnosed with histoplasmosis and immunoglobulin (IgM) deficiency. There has been a suggested association with IgM deficiency and predisposition to bacterial, viral, and fungal infections [2]. We report a case of histoplasmosis in an IgM-deficient individual.

## 2. History

This is the case of a 19-year-old male who presented to the emergency department in a New York hospital with complaints of a nonproductive cough of one-month duration. On review of systems, he admitted a 25-pound weight loss, decreased appetite, subjective fevers with night sweats, decreased exercise tolerance, and pleuritic chest pain. He denied any nausea, vomiting, abdominal pain, rashes, headaches, recent travel, any sick contacts, and recent spelunking or contact with animals. He denied any other medical or surgical history, and he was not taking any home medications. He admitted occasional use of marijuana but otherwise denied any toxic habits.

## 3. Physical Exam

On initial presentation, the patient was afebrile, normotensive, slightly tachycardic, and not in respiratory distress with an oxygen saturation of 97%. He was alert and oriented to name, place, and time. He had no lymphadenopathy, rash, and oral thrush, but his oral mucosa was dry. On examination of his chest, there were bronchial breath sounds noted over the right mid to lower lung zones, no crackles, and no wheezing. Cardiovascular, abdominal, and neurological exams were all otherwise unremarkable.

## 4. Investigations and Treatment

Initial bloodwork was notable for leukocytosis with a white blood cell count (WBC) of 31 × 10^9^/L with neutrophilic predominance 90%, mild normocytic anemia (hemoglobin 12 g/dL and hematocrit 36%), and thrombocytosis thought to be reactive (platelets 505 × 10^9^/L). He had hypoalbuminemia (2.88 g/L). Basic metabolic panel showed normal kidney function (bicarbonate of 26 mmol/L). Arterial blood gas on room air showed respiratory alkalosis with metabolic alkalosis, normal lactate, and normal alveolar-arterial gradient, adjusted for age. Urine toxicology was positive for marijuana.

**Figure 1:**
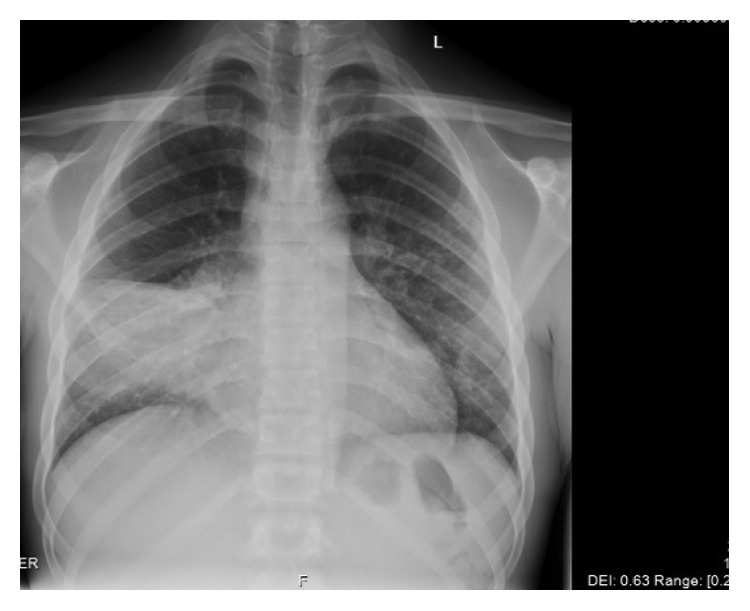
CXR AP frontal view on the day of admission showing right middle lobe infiltrate.

**Figure 2:**
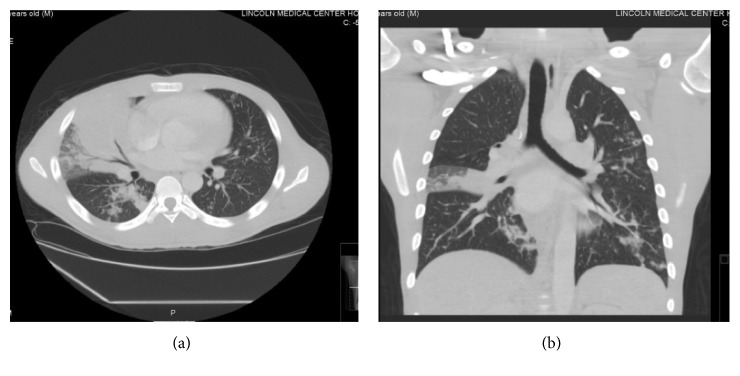
(a) CT chest (axial view) on the day of admission showing the right middle lobe consolidation with a possible necrotic medial segment and enlarged right hilar lymph nodes. (b) CT chest (coronal view) on the day of admission.

Radiographically, his chest X-ray showed a right middle lobe infiltrate (Figure 1), prompting a CT of the chest which confirmed the right middle lobe consolidation with a possible necrotic medial segment and enlarged right hilar lymph nodes (Figures 2(a) and 2(b)). The patient was started empirically on treatment of community-acquired pneumonia with ceftriaxone and azithromycin.

**Figure 3:**
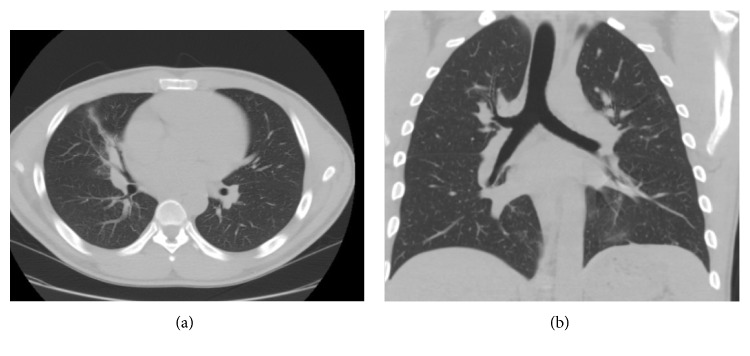
(a) CT chest (axial view) three months after initiation of treatment showing near-complete resolution of the right middle lobe infiltrate. (b) CT chest (coronal view) three months after initiation of treatment.

Despite treatment, the patient had persistent leukocytosis as high as 35 × 10^9^/L (WBC) and began developing high-grade fevers on day 3. The atypical clinical presentation as well as the failure of response to antibiotics prompted a pulmonary evaluation for bronchoscopy. Cytology of the transbronchial biopsy showed chronically inflamed benign bronchial tissue as well as alveolar lung tissue with reactive pneumocytes and poorly formed granulomas. Fungal and acid-fast staining was negative. The urine histoplasma antigen was detected. Given the high specificity of urine histoplasma, the patient was started on IV amphotericin B for a total of nine days. After showing substantial clinical improvement, he was switched to oral Itraconazole. An immunological workup was sent which revealed negative human immunodeficiency virus (HIV) testing and lymphoma/leukemia evaluation but uncovered selective IgM deficiency (<0.20 g/L) with otherwise normal immunoglobulin G (IgG), immunoglobulin A (IgA), immunoglobulin E (IgE), and hemolytic complement 50 (CH50) levels. He was discharged after marked clinical improvement and has followed since with immunology, pulmonary, and infectious disease clinics. His repeat CT scan of lungs showed near-complete resolution of the right middle lobe consolidation (Figures 3(a) and 3(b)). His IgM levels were repeated as outpatient confirming the deficiency. No evidence of malignancy or manifestations of autoimmune disorders was identified.

## 5. Discussion

Histoplasmosis is a dimorphic fungus, which grows in the form of yeast in infected tissues. Infection is largely caused by inhalation of droppings from infected birds or bats. *Histoplasma capsulatum* mainly infects the lungs and passes asymptomatically to involve the skin and reticuloendothelial system [1]. In immunocompetent individuals without underlying structural lung disease, the infection is usually benign and asymptomatic. Rarely, serious or fatal disease may develop usually from other preexisting disease in an abnormal host with compromised immunity [3].

Human infections caused by *Histoplasma capsulatum* may present as acute pulmonary histoplasmosis, chronic pulmonary histoplasmosis, and progressive disseminated histoplasmosis. Patients with underlying lung disease are specifically at risk for developing chronic pulmonary histoplasmosis, which may resemble reactivation of tuberculosis in its clinical presentation and radiological appearance [4]. Our case involved a young patient with chronic pulmonary histoplasmosis and selective IgM deficiency but without underlying lung disease.

IgM is the first immunoglobulin isotype expressed on the cell surface of immature B cells during B-lymphocyte lineage differentiation and represents the first antibody that is produced during an immune response after initial antigen encounter [5]. Circulating human polyclonal IgM is present in plasma at a concentration approximately between 0.5 and 2.0 g/L in healthy adults, with a half-life of about 5 days [6].

Primary selective IgM deficiency is thought to be a rare primary immunodeficiency disease. The prevalence ranges from 0.03 to 3.80% in various studies. It is characterized by low serum level of IgM (<0.20 g/L) and normal IgG and IgA levels. The IgE levels can be increased [7]. The pathogenesis of selective IgM deficiency is unclear. However, decreased T-helper activity, increased isotype-specific suppressor T-cell activity, and intrinsic B-cell defects have been reported, predisposing those patients to infections with extracellular and intracellular bacteria, viruses, and fungi [2, 8].

Rheumatologic and autoimmune disorders such as celiac disease, systemic lupus erythematosus (SLE), and Hashimoto thyroiditis have been reported in association with selective IgM deficiency. In some cases, treatment of the associated autoimmune disorder led to normalization of the IgM levels, whereas others showed no change after treatment [9, 10]. Furthermore, recent experimental studies reported an increased incidence of spontaneous development of IgG anti-DNA antibodies in lupus-prone mice models with selective IgM deficiency [11].

The pathogenesis of this association remains unclear. One theory suggests that IgM has a protective role against autoimmunity by clearing apoptotic cells. Apoptotic cells are potential immunogens that trigger the development of autoimmunity since they express autoantigens such as nucleosomes, DNA, and histones on their surface [12, 13]. It was also hypothesized that one of the first cellular events in SLE is increased apoptosis of certain cells that triggers autoimmunity. This is further supported by the observation that lupus patients have a higher number of apoptotic cells that release oligonucleosomes, and that antinucleosome antibodies are detected before anti-DNA antibodies in patients with SLE [12, 14, 15].

## 6. Conclusion

This case serves as an important reminder to broaden the differential diagnosis in presumably immunocompetent patients who present with pneumonia to include fungal diseases such as pulmonary histoplasmosis, especially if there is poor response to broad-spectrum antibiotics initially. Secondly, this case points out the absolute necessity of screening the patients diagnosed with fungal pulmonary disease for immunological dysfunction. Given the literature, patients with selective IgM deficiency may have a predisposition to certain fungal infections [2]. If indeed IgM deficiency is established in such patients, they should be screened for autoimmunity given recent research [12, 13]. A greater awareness of the link between fungal infections and IgM deficiency may facilitate earlier diagnosis, closer outpatient monitoring, and a potential to discover underlying autoimmune diseases. Finally, if encouraged to report future cases of fungal infections, IgM deficiency and any associated autoimmune conditions, larger scale studies may subsequently be pursued.

## Abbreviations

HIV: Human immunodeficiency virus
IgG: Immunoglobulin G
IgA: Immunoglobulin A
IgE: Immunoglobulin E
CH50: Hemolytic complement 50
IgM: Immunoglobulin M
SLE: Systemic lupus erythematosus
WBC: White blood cell count.
